# Bioluminescence Sensing in 3D Spherical Microtissues for Multiple Bioactivity Analysis of Environmental Samples

**DOI:** 10.3390/s22124568

**Published:** 2022-06-17

**Authors:** Maria Maddalena Calabretta, Denise Gregucci, Tiziana Guarnieri, Marina Bonini, Elisa Neri, Martina Zangheri, Elisa Michelini

**Affiliations:** 1 Department of Chemistry “Giacomo Ciamician”, Alma Mater Studiorum—University of Bologna, Via Selmi 2, 40126 Bologna, Italy; maria.calabretta2@unibo.it (M.M.C.); denise.gregucci2@unibo.it (D.G.); martina.zangheri2@unibo.it (M.Z.); 2 Center for Applied Biomedical Research (CRBA), Azienda Ospedaliero-Universitaria Policlinico S. Orsola-Malpighi, 40138 Bologna, Italy; 3 Cell Physiology Laboratory, Department of Biological, Geological and Environmental Sciences (BiGeA), Alma Mater Studiorum Università di Bologna, 40126 Bologna, Italy; tiziana.guarnieri@unibo.it; 4 Health Sciences and Technologies-Interdepartmental Center for Industrial Research (HST-ICIR), University of Bologna, 40126 Bologna, Italy; 5 ARPAE Emilia-Romagna Laboratorio Multisito, Sede di Bologna, Via F Rocchi 19, 40128 Bologna, Italy; mbonini@arpae.it (M.B.); eneri@arpae.it (E.N.)

**Keywords:** bioluminescence, 3D cell models, microtissue, anti-oxidant activity, inflammatory activity, heavy metals, toxicity

## Abstract

The development of predictive *in vitro* sensing tools able to provide rapid information on the different bioactivities of a sample is of pivotal importance, not only to monitor environmental toxicants, but also to understand their mechanisms of action on diverse molecular pathways. This mechanistic understanding is highly important for the characterization of toxicological hazards, and for the risk assessment of chemicals and environmental samples such as surface waters and effluents. Prompted by this need, we developed and optimized a straightforward bioluminescent multiplexed assay which enables the measurement of four bioactivities, selected for their relevance from a toxicological perspective, in bioluminescent microtissues. The assay was developed to monitor inflammatory, antioxidant, and toxic activity, and the presence of heavy metals, and was successfully applied to the analysis of river water samples, showing potential applicability for environmental analyses. The assay, which does not require advanced equipment, can be easily implemented in general laboratories equipped with basic cell culture facilities and a luminometer.

## 1. Introduction

Cell-based sensing represents a promising alternative to other biosensing techniques as it enables the acquisition of highly valuable information concerning bioactivity, toxicity, and bioavailability. Living cells possess the innate ability to respond to external stimuli via the expression of target genes [1,2]. This behavior can be exploited using synthetic biological techniques to introduce reporter genes, for which expression can be easily quantified. Several microbial reporters have been suggested for detection of analytes of environmental, agro-food, forensic, and clinical interest [3,4,5]. Compared to microbial cells, the response of mammalian cells is closer to physiological behavior observed in humans and animals. Recently, the possibility of performing assays using cells grown by means of 3D cell models has opened new potential to improve the predictivity of the information.

Owing to their superior predictivity, three-dimensional (3D) cell models are increasingly replacing conventional 2D cell cultures. 3D cell models, including spheroids, organoids, and microtissues, enable reconstitution of the extracellular matrix and cell–cell interactions, creating an architecture that faithfully reflects the native morphology of organs and tumors [6,7]. In recent years, 3D cellular models have shown suitability as highly predictive bioassays to identify potentially bioactive molecules interacting with molecular targets, representing a very promising and reliable tool for effect-based analysis [8,9]. 3D cell models can be obtained through various methods. Several approaches rely on the use of scaffolds such as hydrogels and porous substrates on which the cells grow [10]. The standardization of 3D cell models, especially in terms of size and shape, appears to be a vital factor in obtaining robust and reproducible results.

Bioluminescent (BL) reporter assays represent the gold standard for the high-throughput screening assays employed in drug discovery [9,11,12,13]. BL is an optical readout platform widely used in bioassays, with the main advantages of providing a wide dynamic range with high sensitivity, versatility, and biocompatibility. Several reporter assays that use luciferases are widely employed for monitoring cellular events associated with gene expression [14].

A number of cell-based assays have been reported as suitable to monitor protein–protein interactions within cells or living animals, spanning from reporter gene technology to bioluminescence resonance energy transfer (BRET) techniques [15]. More recently, NanoLuc luciferase (NLuc) has been reported as providing another useful tool for developing highly sensitive BL assays [16]. Besides drug screening, other fields are also benefitting from the high sensitivity of BL reporters and probes. More recently, effect-based analysis has also become an invaluable tool for environmental monitoring and for the assessment of the bioactivities of complex matrices such as agro-food samples [8,13,17,18,19].

**Figure 1:**
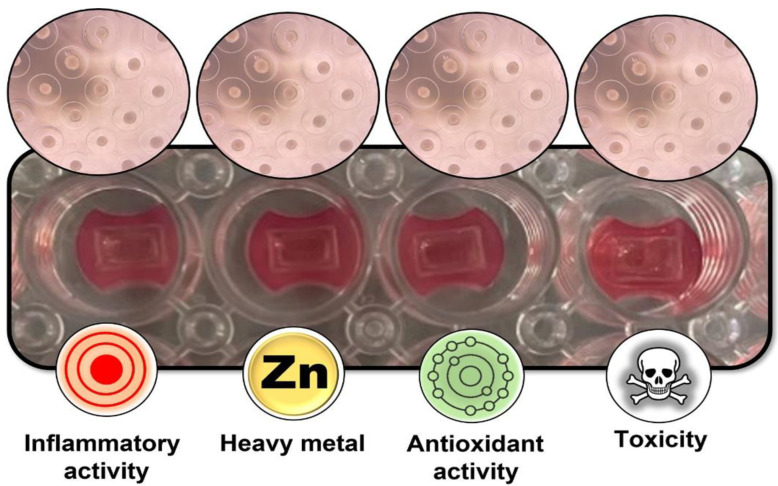
Schematic representation of the bioluminescence sensing platform relying on 3D spherical microtissues for multiple bioactivity analysis.

Whilst several 3D cell models have been reported in the literature, few efforts have been devoted to standardizing BL cell-based assays in 3D formats. Here, we report the development of a new multiplexed assay relying on 3D BL models that enables the acquisition of multiple pieces of information on four different activities via a single assay, without requiring sophisticated instrumentation (Figure 1). The method is capable of investigating four bioactivities in the same assay, with potential applications in several fields. We analyzed river water samples from the Emilia Romagna area, provided by ARPA Emilia-Romagna (the Regional Environmental Protection Agency). The analytical performance of the 3D multiplexed assay was evaluated, and the results confirmed its suitability for accurate and sensitive preliminary assessment of water quality.

## 2. Materials and Methods

### 2.1. Reagents and Plasmids

Human embryonic kidney HEK293T cells were obtained from the American Type Culture Collection (ATCC, Manassas, VA, USA). Penicillin, streptomycin, and cell culture reagents were sourced from Carlo Erba Reagents (Cornaredo, Milano, Italy). Charcoal stripped fetal bovine serum (FBS), was purchased from ThermoFisher Scientific (Waltham, MA, USA). The mammalian expression plasmids pGL4.32[luc2P/NF-κB-RE/Hygro], pGL4.37[luc2P/ARE/Hygro], and pGL4.40[luc2P/MRE/Hygro], carrying Luc2P luciferase under the regulation of NF-kB, antioxidant, and metal transcriptional regulations respectively; plasmid pCDNA3Luc2P; PureYieldTM Plasmid Miniprep System kit; FuGENE^®^ HD transfection reagent; and D-luciferin potassium salt were all sourced from Promega (Promega, Madison, WI, USA). ZnSO_4_·7H_2_O, tert-butylhydroquinone (t-BHQ), Tumor Necrosis Factor-α (TNFα, purity higher than 95%), dimethyl sulfoxide (DMSO), MicroTissues^®^ 3D Petri Dish^®^ micro-mold spheroids, and all other chemicals were purchased from Sigma-Aldrich (St. Louis, MO, USA). River water samples from the Emilia-Romagna area, including transitional (delta and ria), and surface waters, were provided by ARPA Emilia-Romagna (the Regional Environmental Protection Agency).

### 2.2. 2D Assay Format and Transfection

The HEK293T cell line was cultured using DMEM supplemented with 10% (v/v) FBS, 50 U mL^−1^ penicillin, 50 µg mL^−1^ streptomycin, and 2 mM L-glutamine. The day before transfection, the cells were plated into a 96-well optical-bottom black plate at a density of 2.0 × 10^4^ cells/well with 100 µL of complete growth medium, and incubated under standard conditions for 24 h at 37 °C and 5% CO_2_. Afterwards, HEK293T cells were transiently transfected with 0.10 µg of the pGL4.32[luc2P/NF-κB-RE/Hygro], pGL4.37[luc2P/ARE/Hygro], pGL4.40[luc2P/MRE/Hygro] and pCDNA3Luc2P plasmids using the FUGENE^®^ HD transfection reagent at a ratio of 1:3, and incubated for 48 h under the standard conditions. The 2D cell cultures were monitored every 24 h for a period of 3 days using Thermo Scientific Invitrogen Evos M5000 Imaging Systems with a 4× objective.

*Inflammation assay.* HEK293T cells were transiently transfected into a 96-well plate format with a pGL4.32[luc2P/NF-κB-RE/Hygro] construct to monitor inflammatory pathway activation using TNFα as a model compound. After 48 h post transfection in the 2D format, the cell culture medium was replaced with 100 µL of fresh medium containing TNFα (concentration range from 0.1 to 20.0 ng/mL), and incubated for 5 and 24 h at 37 °C with 5% CO_2_. BL measurements (20 min with 1000 ms integration time) were obtained from the 96-well plates using a Tecan Microplate Reader Spark^®^ (Tecan Trading AG, Männedorf, Switzerland) after injection of 100 μL of 1.0 mM D-luciferin citrate solution, pH 5.0.

*Antioxidant assay*. HEK293T cells transiently transfected with a pGL4.37[luc2P/ARE/Hygro] construct were used to monitor antioxidant pathway activation with t-BHQ, which was selected as a model compound to induce nuclear factor erythroid 2–related factor 2 (Nrf2). After 48 h post transfection in the 96-well plate (2D format), the cell culture medium was replaced with 90 µL of DMEM with 0.5% v/v of charcoal stripped FBS. After 24 h, the cells were treated in triplicate with t-BHQ (concentration range from 0.1 µM to 1.0 mM), by adding 10 µL t-BHQ per well and incubating for 5 and 24 h at 37 °C with 5% CO_2_. BL measurements were obtained using a Tecan Microplate Reader Spark^®^ after injection of 100 μL of 1.0 mM D-luciferin citrate solution, pH 5.0.

*Metal activation*. pGL4.40[luc2P/MRE/Hygro] plasmid was used to transiently transfect HEK293T cells to monitor metal pathway activation using ZnSO_4_·7H_2_O. After 48 h post transfection in the 2D format, the HEK293T cell media in the 96-well plate was replaced with 90 µL of DMEM containing 0.5% v/v charcoal stripped FBS, and the cells were incubated for 24 h. Then, the cells were exposed in triplicate to increasing final concentrations (from 0.01 to 100 µM) of the metal inducer, by adding a volume of 10 µL ZnSO_4_ solution (concentration range from 0.1 to 1000 µM) per well, and subsequently incubating for 5 and 24 h at 37 °C with 5% CO_2_. BL measurements were obtained using a Tecan Microplate Reader Spark^®^ after injection of 100 μL of 1.0 mM D-luciferin citrate solution, pH 5.0.

*Toxicity assessment*. HEK293T cells were transiently transfected with pCDNA_Luc2P for toxicity assessment using TNFα, t-BHQ, and ZnSO_4_ as analytes. After 48 h post transfection in the 2D format, the cells were treated with TNFα (concentration range from 0.1 to 20.0 ng/mL), t-BHQ (concentration range from 0.01 µM to 100 µM), and ZnSO_4_ solution (concentration range from 0.1 to 100 µM), and incubated for 5 and 24 h at 37 °C with 5% CO_2_. BL measurements (20 min with 1000 ms integration time) were obtained from the 96-well plates using a Tecan Microplate Reader Spark^®^ (Tecan Trading AG, Männedorf, Switzerland) after injection of 100 μL of 1.0 mM D-luciferin citrate solution, pH 5.0.

### 2.3. Fabrication of Hydrogels, 3D Microtissues, and Transfection

The HEK293T cell line was cultured using DMEM supplemented with 10% v/v FBS, 50 U mL^−1^ penicillin, 50 µg mL^−1^ streptomycin, and 2 mM L-glutamine. The day before transfection, cells were plated in flat-bottom, clear, 24-well plates at a density of 8.0 × 10^4^ cells/well with 500 µL of complete growth medium, and incubated under standard conditions for 24 h at 37 °C and 5% CO_2_. Then, the HEK293T cells were transiently transfected with 0.50 µg of either pGL4.32[luc2P/NF-κB-RE/Hygro], pGL4.37[luc2P/ARE/Hygro], pGL4.40[luc2P/MRE/Hygro], or pCDNA3Luc2P plasmids according to the manufacturer’s instructions, using a FuGENE HD/DNA ratio of 3:1, and subsequently incubated at 37 °C with 5% CO_2_. The day after transfection, 3D spherical microtissues were generated through self-assembly in a non-adhesive agarose gel, with cylindrical microwells created using a 5 × 7 array micro-mold with 800 μm diameter rounded pegs fitting 24 well-plates (3D Petri Dish^®^, MicroTissues Inc., St. Louis, MO, USA). Briefly, a 330 µL-volume of a 2% w/v agarose solution in 0.9% w/v NaCl was pipetted into the micro-mold (3D Petri Dish, MicroTissues Inc.). After the agarose had gelled, the agarose gel was separated from the micro-mold and transferred to a single well of a clear 24-well plate. To equilibrate the 3D Petri Dish^®^, 1.0 mL of fresh cell culture medium was added per well, and then after 15 min incubation, replaced with fresh cell culture medium. This procedure was repeated twice, using 10% v/v FBS to monitor inflammatory pathway activation, and 1.0 mL of fresh medium containing 0.5% v/v charcoal stripped FBS to monitor antioxidant and metal pathway activations. Both 10% v/v FBS and 0.5% v/v charcoal stripped FBS were used for the general toxicity assay. Cells previously transfected in the 24-well plate were detached with trypsin, centrifuged for 5 min at 1200 rpm, resuspended in fresh medium, counted to a density of 3.5 × 10^4^ in 75 µL, and transferred to micro-molds placed in the 24-well clear plate. After 10 min, to allow the cells to settle into the micro-mold, 1.0 mL of complete medium was added to each well. Spheroid formation was attained by incubating the plate at 37 °C and 5% CO_2_. Spherical microtissues were monitored every 24 h for a period of 7 days using the Thermo Scientific Invitrogen Evos M5000 Imaging Systems with a 4× objective.

### 2.4. 3D Bioluminescence Sensing for Multiple Bioactivity Assay

For the 3D bioactivity multiplexed assay, we used one-day-old HEK293T spheroids from the 3D Petri Dish^®^ already transfected with the corresponding plasmids (as described in Section 2.3). For the inflammatory bioactivity test, the cell culture medium was replaced with 1 mL of fresh medium containing TNFα (concentration range from 0.1 to 20.0 ng/mL). The same procedure was carried out with HEK293T cells transiently transfected with pCDNA_Luc2P for toxicity assessment. As an analyte model for simulated toxicity, we used DMSO (concentration range from 0.01 to 20% v/v), TNFα (concentration range from 0.1 to 20.0 ng/mL), t-BHQ (concentration range from 0.01 µM to 100 µM), and ZnSO_4_ (concentration range from 0.01 µM to 100 µM). For the antioxidant 3D format assay, the same procedure was used with 0.5% v/v of charcoal stripped FBS, and 120 µL t-BHQ solution in H_2_O (concentration range from 0.1 µM to 1.0 mM) was added to each well. For the metal pathway activation, 120 µL ZnSO_4_ solution (0.1 to 1000 µM) was added to the medium.

**Figure 2:**
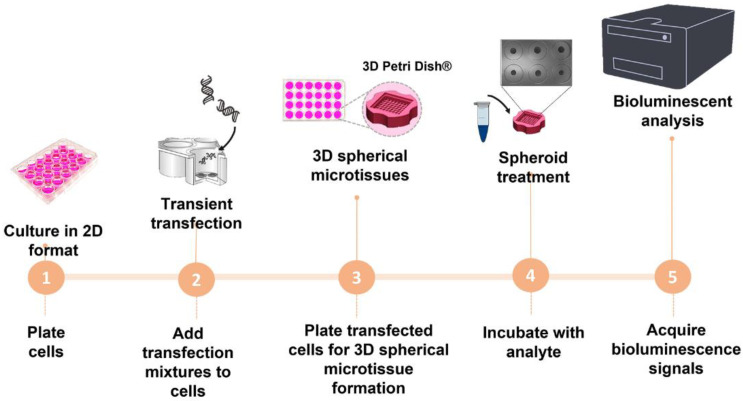
Schematic representation of the acquisition of the BL 3D spherical microtissues for monitoring inflammatory and antioxidant bioactivities, presence of heavy metals, and toxicity.

After incubation for 5 h and 24 h at 37 °C with 5% CO_2_, 1 mL and 50 μL aliquots of the media was removed from outside and inside the mold, respectively. A 50 μL volume of 1.0 mM D-luciferin citrate solution, pH 5.0, was added to the inside of the mold, and BL kinetics (20 min with 1000 ms integration time, Figure S1) were obtained with a Tecan Microplate Reader Spark^®^ (Figure 2).

### 2.5. Real Samples

The suitability of the 3D BL high-content sensing technique to provide information about pro-inflammatory activity, antioxidant activity, general toxicity, and presence of heavy metals in environmental samples was investigated. To this end, river water samples from the Emilia-Romagna area were used to simulate a complex matrix with multiple bioactivities. Samples were spiked with ZnSO_4_, t-BHQ, and TNFα at final concentrations of 50 µM, 10 µM, and 5 ng/mL, respectively. One-day-old HEK293T spheroids from the 3D Petri Dish^®^ transfected with the corresponding plasmids (as described in Section 2.3) were used to test for inflammatory and antioxidant bioactivities, the presence of heavy metals, and toxicities. Six river water samples (named BS1, BS2, BS3, BT1, BT2, and BT3) spiked with ZnSO_4_ (50 µM), t-BHQ (10 µM), and TNFα (5 ng/mL) were tested (samples S1, S2, S3, T1, T2, and T3). Each sample was tested three times and in triplicate. A 120 µL volume of spiked river water sample was added per well, and the plates incubated for 5 h at 37 °C with 5% CO_2_; then 1 mL and 50 μL aliquots of the media was removed from outside and inside the mold, respectively. A 50 μL volume of 1.0 mM D-luciferin citrate solution, pH 5.0, was added to the inside of the mold, and the BL kinetics (20 min with 1000 ms integration time) were measured. BL responses were expressed as fold-changes relative to the control by dividing the BL signal of cells treated with spiked river sample (S1, S2, S3, T1, T2, and T3) by the corresponding control (BS1, BS2, BS3, BT1, BT2, and BT3).

### 2.6. Statistical Analysis

The BL bioassay responses were expressed as fold-changes relative to the control by dividing the BL signal of the treated cells by the corresponding control (either cell culture medium or ddH_2_O). TNFα, t-BHQ, and ZnSO_4_ dose-response curves in 2D and 3D formats were generated using GraphPad Prism v8.3.0 software (GraphPad Software, La Jolla, CA, USA), and the data were plotted normalizing the BL signal in respect to the control. The limit of detection (LOD) was calculated as the inducer that corresponded to the blank plus three times the standard deviation (s.d.).

The half maximal effective concentration (EC_50_), which is the concentration of the inducer which produces 50% of the maximum possible response, was calculated using the following equation:

Y = Bottom + (Top − Bottom)/(1 + 10^((LogEC50 − X) × Hillslope))

where X is the logarithmic concentration of TNFα, t-BHQ, or ZnSO4; and Y is the response.

The half maximal inhibitory concentration (IC_50_), which is the concentration of the inhibitor needed to inhibit a biological process or response by 50%, was calculated using the following equation:

Y = Bottom + (Top − Bottom)/(1 + 10^((X − LogIC50) × Hillslope))

where X is the logarithmic concentration of DMSO, and Y is the response.

All measurements were performed in triplicate and repeated at least three times. A one-tailed *t*-test was applied to the real samples analysis data for inflammatory, antioxidant, and metal pathway activations using using GraphPad Prism v8.3.0 software (GraphPad Software, La Jolla, CA, USA).

## 3. Results

### 3.1. 3D Bioluminescence Microtissues

**Figure 3:**
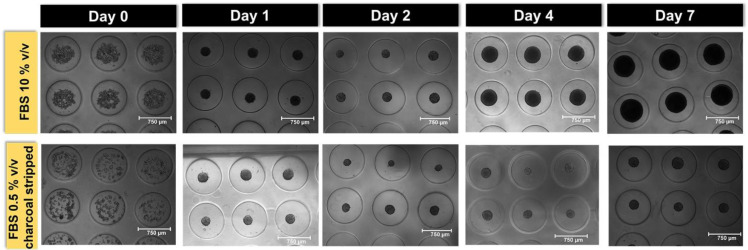
Growth monitoring of 3D spherical microtissues in 10% v/v FBS and 0.5% v/v charcoal stripped FBS. Brightfield images were acquired with Thermo Scientific Invitrogen Evos M5000 Imaging Systems using a 4× objective.

In order to develop a multiplexed assay for monitoring four different activities in the same sample, we first optimized the conditions to obtain spherical microtissues with a defined shape and dimensions using a reproducible protocol which can be easily implemented in other laboratories. Figure 3 shows the microtissues in the micro-mold on different days after cell seeding employing cell culture medium supplemented with either 10% v/v FBS or 0.5% v/v charcoal stripped FBS. After 2 days, the average diameter of the microtissues was 240 ± 20 µm or 210 ± 10 µm for cells grown in 10% v/v FBS or in 0.5% v/v charcoal-stripped FBS, respectively.

### 3.2. 3D Multiple Bioactivity Assay for Inflammatory and Oxidative Pathway Activations, Toxicity, and Presence of Heavy Metals

One-day-old HEK293T microtissues, having an average diameter of 180 ± 20 µm, and previously transfected in the 2D monolayer with the reporter constructs, in which the Luc2P luciferase was placed under the control of the NF-kB, ARE, MRE, or CMV promoter, were incubated in the micro-molds with the different analytes, i.e., TNFα (concentration range of 0.1–20 ng mL^−1^), t-BHQ (concentration range of 0.01–100 µM), ZnSO_4_ (concentration range of 0.01–100 µM), or DMSO (concentration range of 0.01–20% v/v), for 5 and 24 h at 37 °C. The BL emission kinetics of the 3D cell cultures were obtained after the addition of 50 µL of D-luciferin substrate 1.0 mM, pH 5.0, and showed a BL signal maximum between 30 and 60 s (Figure S1).

**Figure 4:**
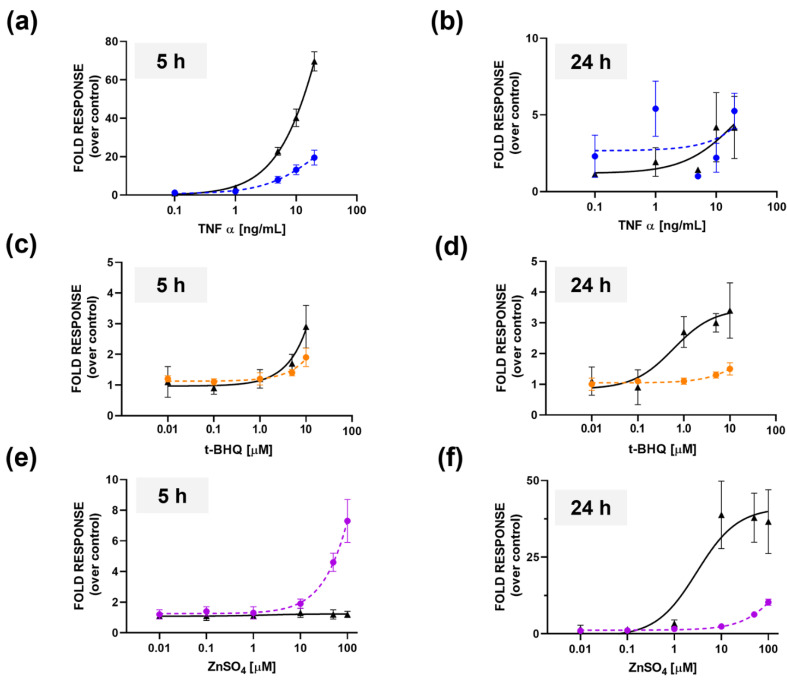
Dose-response curves obtained for 2D (solid line) and 3D cultures (dotted line) incubated with TNF-α at 5 h (**a**) and 24 h (**b**); with t-BHQ at 5 h (**c**) and 24 h (**d**); and with ZnSO_4_ at 5 h (**e**) and 24 h (**f**).

To investigate the inflammation activity, dose-response curves for TNFα with 5 and 24 h incubation periods were obtained, and compared with dose-response curves obtained from the 2D monolayer cultures. The LODs were 0.41 and 0.54 ng/mL for 2D cell cultures and microtissues, respectively, at 5 h incubation; and EC_50_ values of 50.1 ± 0.2 and 22.7 ± 0.3 ng mL^−1^ were obtained for the 2D and 3D formats, respectively (Figure 4a). Following 24 h of incubation, a LOD of 0.02 ± 0.01 ng mL^−1^ and an EC_50_ of 22.7± 0.02 ng mL^−1^ were obtained for the monolayer cultures; while for the microtissues it was not possible to calculate the values due to low BL intensities (Figure 4b).

**Figure 5:**
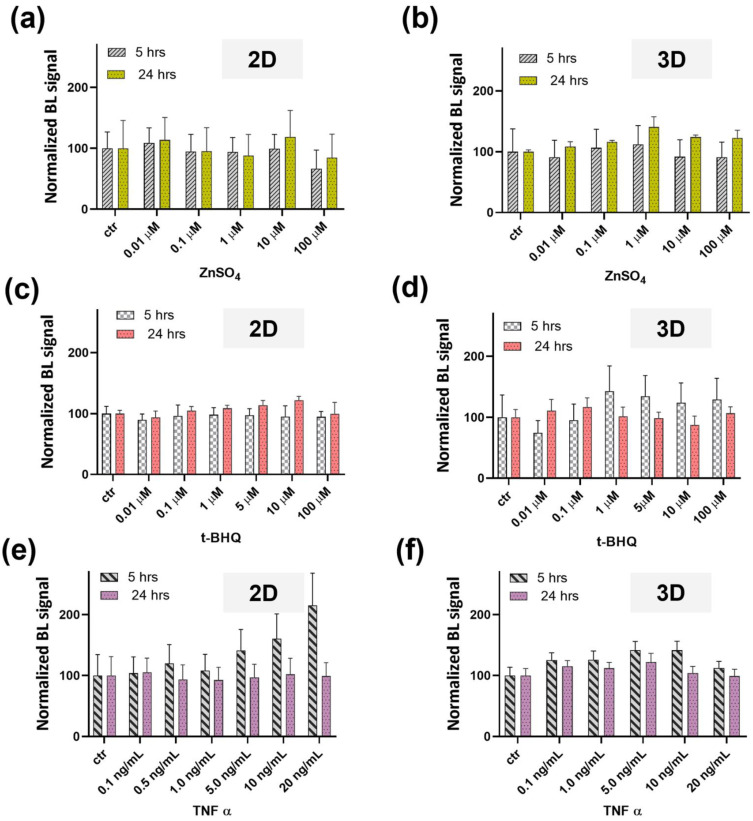
Toxicity curves obtained for 2D and 3D cell cultures grown in FBS 10% v/v medium and transfected with pCDNALuc2P. 2D cell cultures (**a**) and 3D microtissues (**b**) incubated with ZnSO_4_ solutions (concentration range from 0.01 to 100 µM) for 5 h and 24 h. 2D cell cultures (**c**) and 3D spherical microtissues (**d**) incubated for 5 h and 24 h with t-BHQ solutions (concentration range from 0.01 to 100 µM). 2D cell cultures (**e**) and 3D microtissues (**f**) incubated with TNFα (from 0.1 to 20 ng/mL) for 5 h and 24 h.

To elucidate antioxidant activity, transfected microtissues were incubated with t-BHQ (final concentration range from 0.01 to 100 µM) for 5 h and 24 h. Spheroids treated with 10 µM t-BHQ showed approximately a 1.9-fold increase in luciferase activity compared to the control (incubated with ddH_2_O) (Figure 4c). A threefold increase was also observed in 2D cells treated with 10 µM t-BHQ (Figure 4c). This increase in activity was not detected in cells grown in culture medium with 0.5% v/v charcoal stripped FBS treated with 100 µM t-BHQ; this was also confirmed by morphological analysis (Figure S6). Figure 5 shows the results of the toxicity assessments obtained with HEK293T cells transiently transfected with pCDNA_Luc2P in 2D and 3D formats. The same treatment was repeated for 3D spherical microtissues grown in 0.5% v/v charcoal stripped FBS and higher toxicity was reported at 5 h, corresponding to a 94% reduction in the signal (Figure S2b).

LOD values of 2.40 ± 0.2 μM and 5.1 ± 0.2 μM were obtained after 5 h incubation periods in the 3D and 2D formats, respectively (Figure 4c). After 24 h of incubation, LODs of 25.3 ± 1.2 μM and 0.4 ± 0.1 μM were obtained for the 3D and 2D formats, respectively (Figure 4d). An EC_50_ value of 0.6 ± 0.1 μM was observed in the 2D format after 24 h of treatment with t-BHQ. Despite the 2D cultures providing a higher dynamic range and sensitivity at 24 h, we observed a higher variability between replicates than that observed with the 3D spherical microtissues. This was confirmed by growth monitoring of 2D cell cultures in 10% v/v FBS and 0.5% v/v charcoal stripped FBS (Figure S3).

To test the activation of the MTF-1-MRE pathway through a zinc dependent mechanism, one-day-old HEK293T spheroids, transfected with pGL4.40[luc2P/MRE/Hygro] plasmid, were treated with ZnSO_4_ (final concentration range from 0.1 to 100 μM). After 5 h of incubation time, a 6.1-fold increase in BL was observed in the 3D spherical microtissue treated with 100 μM ZnSO_4_, compared to the 2D format (Figure 4e). Conversely, after 24 h, a 3.6-fold increase was observed in the 2D format compared to the 3D spherical microtissues (Figure 4f). LOD values of 4.7 ± 0.3 and 0.86 ± 0.02 μM were obtained with spheroids at 5 (Figure 4e and Figure S8) and 24 h (Figure 4f), respectively, while for the 2D format a LOD of 0.23 ± 0.03 μM and an EC_50_ of 3.0 ± 0.1 was obtained at 24 h (Figure 4e).

In relation to the toxicity assessments, microtissues previously transfected with pCDNALuc2P, and grown in 10% v/v FBS medium, were incubated with ZnSO_4_ (final concentration range from 0.01 to 100 µM), t-BHQ (final concentration range from 0.01 to 100 µM), and TNFα (final concentration range 0.1–20 ng/mL) for 5 and 24 h (Figure 5). Five-hour treatment with ZnSO_4_ at the tested concentrations did not result in significant toxicity; at 24 h only a significant BL signal increase of 40% was observed with 1.0 μM ZnSO_4_ (Figure 5b). Repeating the same treatment for the 3D spherical microtissues grown in medium with 0.5% v/v charcoal stripped FBS, a BL signal increase of about 30% was observed at 5 and 24 h in the presence of 1.0 μM ZnSO_4_ (Figure S2a). 3D spherical microtissues grown in 10% v/v FBS medium and treated with t-BHQ showed a BL signal increase characterized by higher variability, with a coefficient of variation (CV%) of 25% (Figure 5d). We did not observe toxicity with t-BHQ at 100 μM. Conversely, a higher toxicity, corresponding to a significant BL signal decrease of 95% was found in microtissues grown in 0.5% v/v charcoal stripped FBS medium and treated with 100 μM t-BHQ (Figure S2b). An increased BL signal (approximately 35%) was reported in 3D spherical microtissues treated with TNFα (Figure 5f). In addition, in the 2D format, we observed an increased BL signal proportional to the concentration of TNFα, yielding a two-fold higher signal for cells treated with 20 ng/mL TNFα compared to the control (Figure 5e).

In the 3D spherical microtissues, an IC_50_ of 5.8 ± 0.7% v/v was calculated at 5 h after treatment with DMSO (concentration range from 0.1 to 20% v/v using charcoal stripped FBS 0.5% v/v vs. an IC_50_ of 7.3 ± 0.5% v/v obtained with FBS 10% v/v treatment (Figure S4a). At 24 h, DMSO toxicity curves (concentration range from 0.1 to 20% v/v) were obtained, and an IC_50_ of 7.9 ± 0.7% v/v was calculated in 3D spherical microtissues cultured in charcoal stripped FBS 0.5% v/v vs. an IC_50_ of 7.7 ± 0.5% v/v obtained in FBS 10% v/v (Figure S4b).

### 3.3. Real Sample Analysis

**Figure 6:**
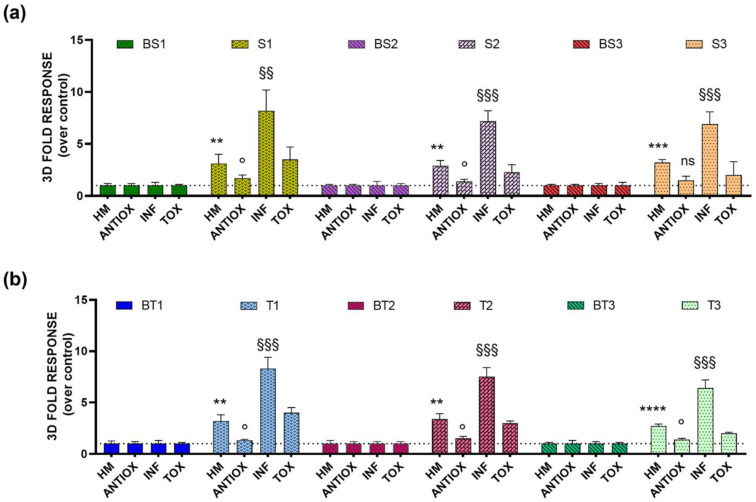
Antioxidant (ANTIOX) and inflammatory (INF) bioactivities, heavy metals presence (HM) and toxicity (TOX) of (**a**) superficial (BS1, BS2, and BS3) and spiked superficial (S1, S2, and S3) river samples; (**b**) transitional (BT1, BT2, and BT3) and spiked transitional (T1, T2, and T3) river samples, tested at 5 h in 3D spherical microtissues transfected with pGL4.37[luc2P/ARE/Hygro], pGL4.32[luc2P/NF-κB-RE/Hygro], pGL4.40[luc2P/MRE/Hygro], and pCDNALuc2P, respectively. One-tailed *t*-test was used to calculate statistical significance for the presence of heavy metals: ns = not significant (*p* > 0.05); ** = *p* ≤ 0.01; *** = *p* ≤ 0.001; **** = *p* ≤ 0.0001; for antioxidant activity: ns = not significant (*p* > 0.05); ° = *p* ≤ 0.05; and for inflammatory activity: ns = not significant (*p* > 0.05); ^§§^ = *p* ≤ 0.01; ^§§§^ = *p* ≤ 0.001.

To investigate using our BL 3D high-content sensing method for the predictive and cost-effective characterization of the bioactivity of unknown samples, river water samples were spiked with TNFα, t-BHQ, and ZnSO_4_ to simulate a complex environmental matrix characterized by multiple bioactivities: antioxidant (ANTIOX) and inflammatory (INF) bioactivities, the presence of heavy metals (HM), and toxicity (TOX). Once optimized, we employed the analytical procedure involving the multiplexed assay relying on 3D BL models (Figure 6), to analyse six river water samples (S1, S2, and S3 from superficial river water, and T1, T2, and T3 from transitional river water) spiked with t-BHQ (final concentration 10 µM), TNFα (final concentration 5 ng/mL), and ZnSO_4_ (final concentration 50 µM), and incubated them at 37 °C for 5 h. Both the spiked superficial and transitional river samples showed a significant increase in inflammatory activity—approximately 5 times higher than the control treated with the same concentration of TNFα (5 ng/mL).

## 4. Discussion

The development of predictive *in vitro* sensing tools able to provide rapid information about various bioactivities of a sample is of pivotal importance, not only to monitor environmental toxicants, but also to understand their mechanisms of action on different molecular pathways. This mechanistic understanding is highly important when characterizing toxicological hazards, and for the risk assessment of chemicals and environmental samples such as surface waters and effluents [20].

Prompted by this need, we developed and optimized a straightforward BL multiplexed assay which enables the measurement of four bioactivities, selected for their relevance from a toxicological perspective, in BL microtissues. The assay, which does not require advanced equipment, can be easily implemented in laboratories equipped with basic cell culture facilities and a luminometer. We chose the HEK293T cell line for its high transfection efficiency and for its capability to rapidly aggregate in 3D microtissues in less than 24 h [8,21,22]. We monitored spheroid formation for 1 week (Figure 2). Since 3D cell models have limited availability of both oxygen and the analytes and substrates required for luciferase-catalysed reactions, we selected microtissues with average diameters of 240 ± 20 µm when grown in 10% v/v FBS, and of 210 ± 10 µm when grown in 0.5% v/v charcoal stripped FBS (Figure 3). In fact, while it has been previously reported that spheroids with a 150 µm diameter have enough oxygen for BL reactions, with only 2% of cells being under hypoxic conditions [23], we did not observe any significant decrease in BL between 240 µm and 150 µm-diameter spheroids. Agarose was selected as the support material because it is widely used as a mold for fabricating self-assembled, scaffold-free tissue spheroids in less than 24 h, due to its excellent biocompatibility and non-cell adhesive properties [24]. Owing to its permeability to gas and small biomolecules, it has been also applied to the evaluation of anti-cancer drugs [25,26].

The assay was developed to monitor inflammatory, antioxidant, and toxic activity, and the presence of heavy metals, with the major goal of upgrading conventional 2D cell-based assays to produce a multiplexed assay that provides quantitative results for four different bioactivities. This enables the acquisition of more comprehensive results with reduced cost and time. To the best of our knowledge, this approach has not yet been reported in the literature, in which most cell-based assays are performed separately using cells grown as a monolayer under standard protocols [4,11].

We selected four reporter plasmids for monitoring the different target bioactivities, and a reporter protein, Luc2P, the destabilized version of *Photinus pyralis* luciferase, to improve the response of the luciferase reporter to small and rapid changes in gene expression, resulting in increased sensitivity. Despite stable transfections being suitable for studies with reporter vectors, the low copy number of stable integrated DNA results in lower levels of protein expression compared with the higher level of protein expression produced in less time by transient transfections [27].

Moreover, an advantageous aspect for applications using 3D cell models is the red-shifting in the BL emissions, which was observed using D-luciferin substrate in citrate buffer (λ_max_ = 623 nm) (Figure S5a). The lysing Bright-Glo™ commercial substrate leads to a red-shifted emission, but this is less pronounced (λ_max_ = 608 nm) (Figure S5b); therefore we decided to use the D-luciferin substrate—also taking into consideration the reagent cost and the ability to perform non-destructive measurements.

To monitor inflammatory activity, we used the pGL4.32[luc2P/NF-κB-RE/Hygro] plasmid which expresses Luc2P under the regulation of the nuclear factor-kappa B (NF-κB) response element. NF-κB is a point of convergence of external and internal inflammation triggers, and thus represents a suitable transcription factor for monitoring pro-inflammatory stimuli. When comparing the dose-response curves for TNFα, which we used as a model analyte, we observed a higher NF-kB basal activation (3.2 ± 0.4-fold) in 3D spheroids than we did in 2D monolayer cultures (Figure 4a). This is consistent with results previously obtained by us [21] and by Jack et al. [28], who reported the presence of intra-spheroid cytokine signaling that induces the NF-kB and JNK pathways. We also reported an increase in the diameter of spheroids caused by TNFα incubation (e.g., 310 ± 20 μm vs. 270 ± 10 μm in non-treated cells after 24 h of incubation with 10 ng/mL TNFα), presumably due to the proliferation of cells (Figure S7), as also reported by Wang et al. in nucleus pulposus (NP) cells [29].

For the anti-oxidant activity experiments, we used pGL4.37[luc2P/ARE/Hygro] plasmid, which carries four copies of an Nrf2-sensitive antioxidant response element (ARE), driving transcription of the reporter Luc2P. The Nrf2-ARE signal transduction pathway contributes to the transcriptional regulation of stress response genes and is activated by cellular stresses [30]. Nrf2 binds to the ARE present within the promoter region of genes encoding for phase II enzymes, resulting in up-regulation of their transcription. These enzymes are involved in defense of the redox insult by chemicals and xenobiotics, actuating as protectors of the cells from damage [31].

The metal-responsive transcription factor-1 (MTF-1)-MRE signal transduction pathway was selected because it plays a critical role in the homeostasis and detoxification of heavy metals. Moreover, it is interconnected with multiple cell stress response pathways, helping to protect the organism against hypoxia and oxidative stress. Upon zinc binding, MTF-1 shuttles to the nucleus where it binds to MRE and interacts with transcription factors (e.g., Sp1) and coactivators (e.g., p300), driving luciferase expression [32].

To preliminarily investigate the suitability of this 3D platform, considering that both ATP and luciferin availability could be limiting factors for the BL reaction according to spheroid size, BL measurements were performed in non-lysing conditions using HEK293T transfected with a pCDNALuc2P construct expressing Luc2P under the regulation of CMV promoter. D-luciferin substrate in citrate buffer (1.0 mM, pH 5.0) was selected as a non-lysing substrate. We selected D-luciferin substrate because of the low cost per reaction in comparison to commercial substrates, and the possibility of performing non-destructive imaging.

To assess toxicity, we decided to include a micro-mold for testing sample toxicity in both 10% v/v FBS and 0.5% v/v charcoal stripped FBS. This was motivated by the fact that we used both media for the reporter plasmids, as suggested by the producers, but in 0.5% v/v charcoal stripped FBS the toxicity was more pronounced, and this was confirmed by morphological studies (Figure S6). For example, a 94% decrease in the BL signal was found after t-BHQ treatment (100 μM) (Figure S2b).

To confirm the suitability of our BL multiplexed assay to measure four bioactivities, we simulated a real sample using superficial and transitional river waters spiked with ZnSO_4_, t-BHQ, and TNFα. Lower antioxidant pathway activations were observed, probably due to dissolved organic matter photoreactions generating hydroxyl radicals in natural waters [33]. A higher activation of NF-kB transcription factor was observed (approximately 5 times higher) in cells treated with spiked river samples; most likely this was due to complex cross-talk among these pathways [34]. This is consistent with results previously obtained [35]; accumulating evidence suggests that many metals are able to affect the activation or activity of NF-κB transcription factor, either in a dose-dependent or cell type-related manner.

## 5. Conclusions

A multiplexed BL sensing assay based on 3D microtissues was reported. The feasibility of the assay was evaluated using HEK293T cells to assess inflammatory and antioxidant activities, the presence of metals, and the toxicity of a sample, using a unique protocol which enables the assessment of the four activities at once. To the best of our knowledge, this is the first bioluminescent assay of its kind, providing a new straightforward strategy to assess multiple bioactivities in a single sample. The method was applied to river water samples spiked with analytes with multiple bioactivities, and it could be helpful in situations in which mixtures of compounds with unknown bioactivities, toxicities, and different modes of action are present, such as in the aquatic environment, thus representing a useful tool for an initial screening.

## Supplementary Materials

The following supporting information can be downloaded at: https://www.mdpi.com/article/10.3390/s22124568/s1, Figure S1: Emission kinetic of 3D spherical microtissue transfected with pCDNALuc2P after the addition of D-luciferin substrate (1.0 mM, pH 5.0); Figure S2: Toxicity dose-response curves obtained in 3D spherical microtissues transfected with pCDNALuc2P, grown in medium with charcoal stripped FBS 0.5% v/v and treated with (**a**) ZnSO4 solutions and with **(b**) t-BHQ solutions for 5hrs and 24 hrs.; Figure S3: Growth monitoring of 2D cell cultures in 10% v/v FBS and 0.5% v/v charcoal stripped FBS; Figure S4: DMSO toxicity curves obtained at (**a**) 5 h and (**b**) 24 h for 3D spherical microtissues; Figure S5: BL emission spectra obtained in 2D and 3D cell Hek293T models with (**a**) D-Luciferin non-lysing substrate 1.0 mM in citrate buffer, pH 5.0, and (**b**) Bright-Glo™ commercial lysing substrate; Figure S6: Brightfield images of one-day-old HEK293T spheroids transfected with pGL4.37_ARE_Luc2P and treated for 5 h with TNFα; Figure S7: Brightfield images of one-day-old HEK293T spheroids transfected with pGL4.32_NFkB_Luc2P and treated for 5 h with TNFα; Figure S8: Brightfield images of one-day-old HEK293T spheroids transfected with pGL4.40_MRE_Luc2P and treated for 5 h with ZnSO_4_.
