# Supplementary material for: Bioluminescence Sensing in 3D Spherical Microtissues for Multiple Bioactivity Analysis of Environmental Samples
Source: Sensors (Basel). 2022 Jun 17;22(12):4568. doi: 10.3390/s22124568 (PMC9229012; doi:10.3390/s22124568)
Supplement: Supplementary file 1 [file sensors-22-04568-s001.zip › sensors-1767740-supplementary.pdf]

Supplementary materials

Bioluminescence Sensing in 3D Spherical Microtissues for Multiple Bioactivity Analysis of Environmental Samples

Maria Maddalena Calabretta [1,2], Denise Gregucci [1,2], Tiziana Guarnieri [3,4], Marina Bonini [5], Elisa Neri [5], Martina Zangheri [1] and Elisa Michelini [1,2,4,*]

[1] Department of Chemistry "Giacomo Ciamician", Alma Mater Studiorum—University of Bologna,
Via Selmi 2, 40126 Bologna, Italy; maria.calabretta2@unibo.it (M.M.C.); denise.gregucci2@unibo.it (D.G.); martina.zangheri2@unibo.it (M.Z.)

[2] Center for Applied Biomedical Research (CRBA), Azienda Ospedaliero-Universitaria Policlinico S. Orsola-Malpighi, 40138 Bologna, Italy

[3] Cell Physiology Laboratory, Department of Biological, Geological and Environmental Sciences (BiGeA), Alma Mater Studiorum Università di Bologna, 40126 Bologna, Italy; tiziana.guarnieri@unibo.it

[4] Health Sciences and Technologies-Interdepartmental Center for Industrial Research (HST-ICIR), University of Bologna, 40126 Bologna, Italy

[5] ARPAE Emilia-Romagna Laboratorio Multisito, Sede di Bologna, Via F Rocchi 19, 40128 Bologna, Italy; mbonini@arpae.it (M.B.); eneri@arpae.it (E.N.)

* Correspondence: elisa.michelini8@unibo.it; Tel./Fax: +39-0512099533

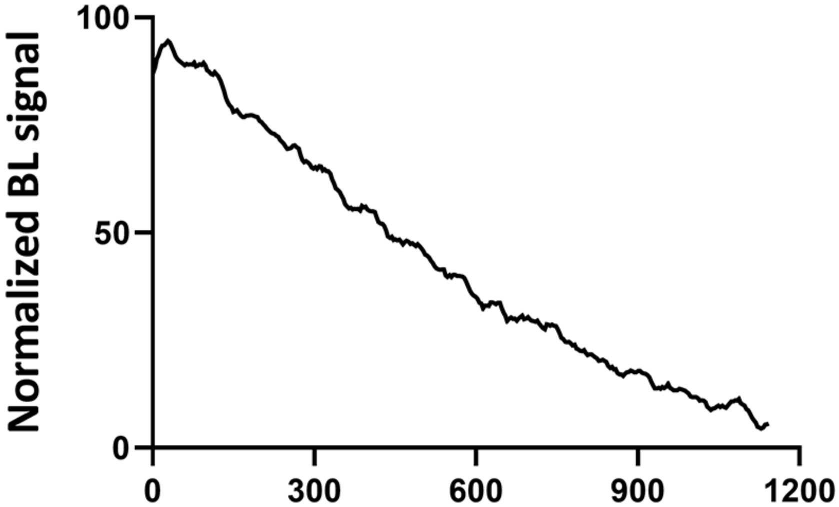

Figure S1: Emission kinetic of 3D spherical microtissue transfected with pCDNALuc2P after the addition of D-luciferin substrate (1.0 mM, pH 5.0).

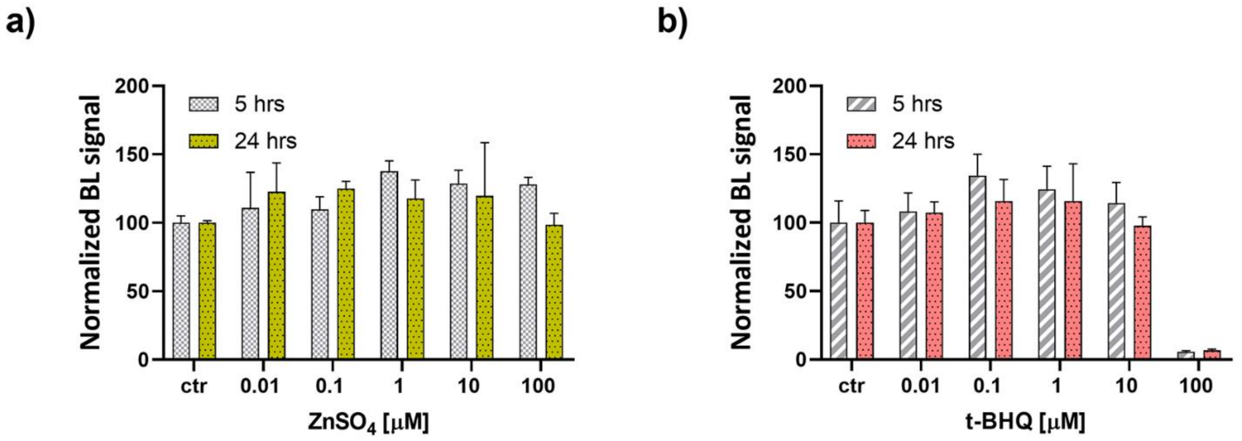

Figure S2: Toxicity dose-response curves obtained in 3D spherical microtissues transfected with pCDNALuc2P, grown in medium with charcoal stripped FBS 0.5% v/v and treated with a) $ZnSO_4$ solutions (concentration range from 0.01 to 100 µM) and with b) t-BHQ solutions (concentration range from 0.01 to 100 µM) (b) for 5hrs and 24 hrs.

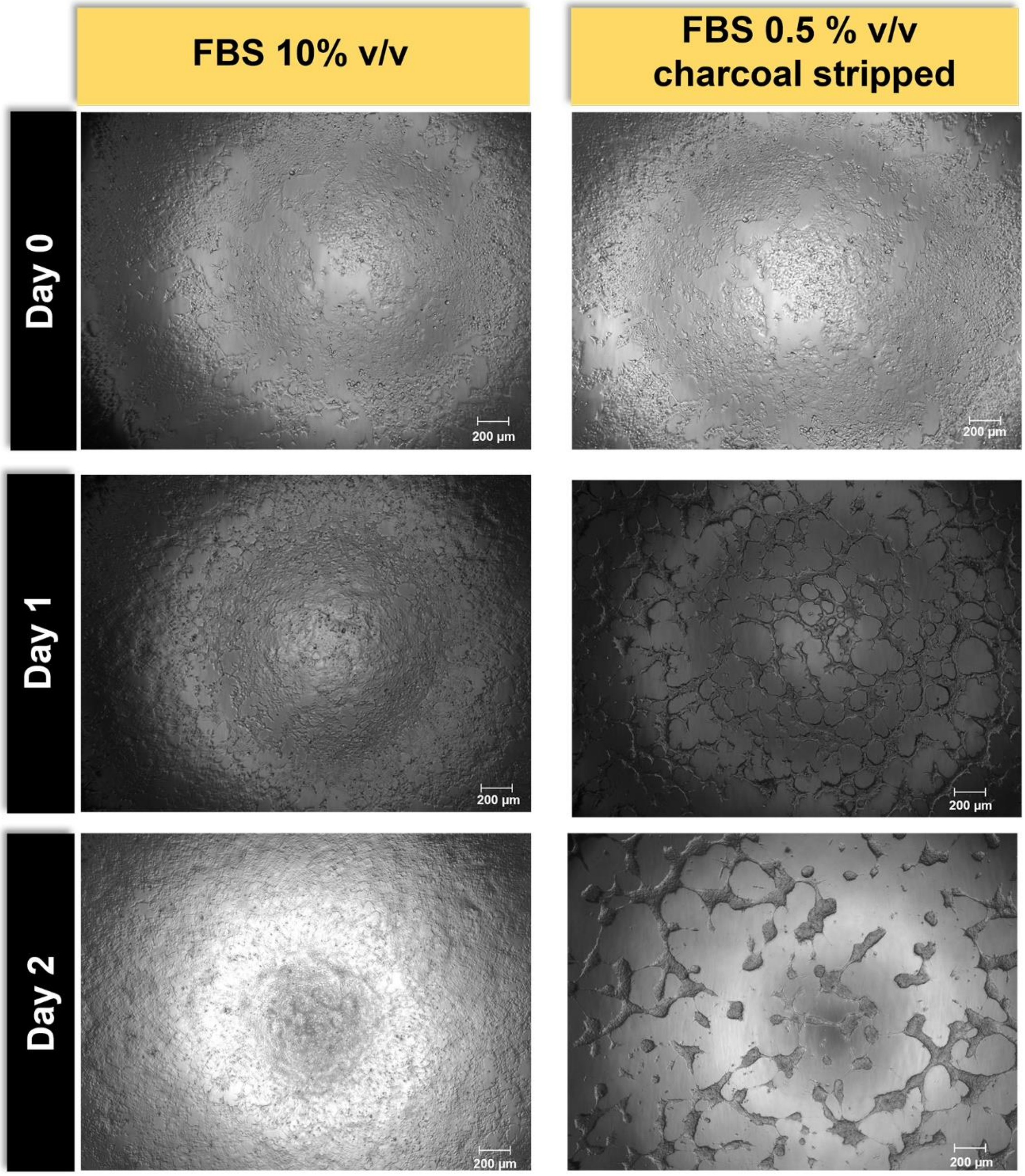

Figure S3: Growth monitoring of 2D cell cultures in 10% v/v FBS and charcoal stripped 0.5% v/v FBS. Brightfield images were acquired with Invitrogen Evos M5000 Imaging Systems Thermo Scientific using an objective 4x

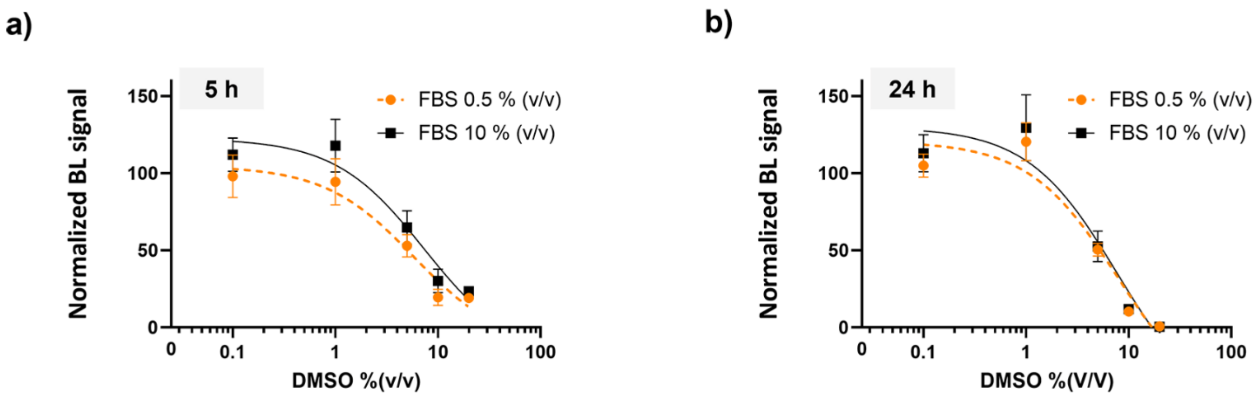

Figure S4: DMSO toxicity curves obtained at a) 5hrs and b) 24 hrs with 3D spherical microtissues cultured in FBS 0.5% (v/v) charcoal stripped and FBS 10% (v/v) medium.

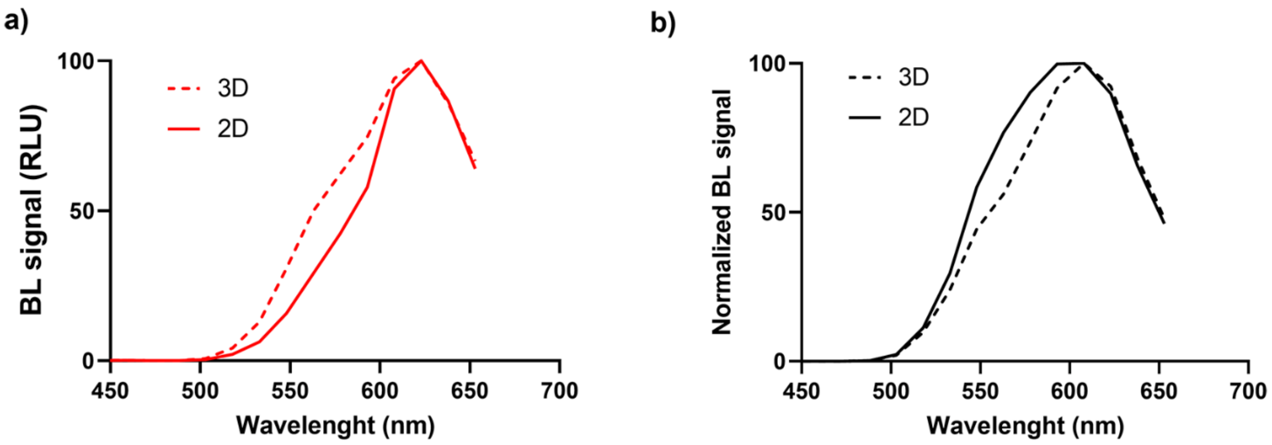

Figure S5: BL emission spectra obtained in 2D and 3D cell Hek293T models with a) D-Luciferin non-lysing substrate 1.0 mM in buffer citrate pH 5.0 and b) Bright-Glo™ commercial lysing substrate.

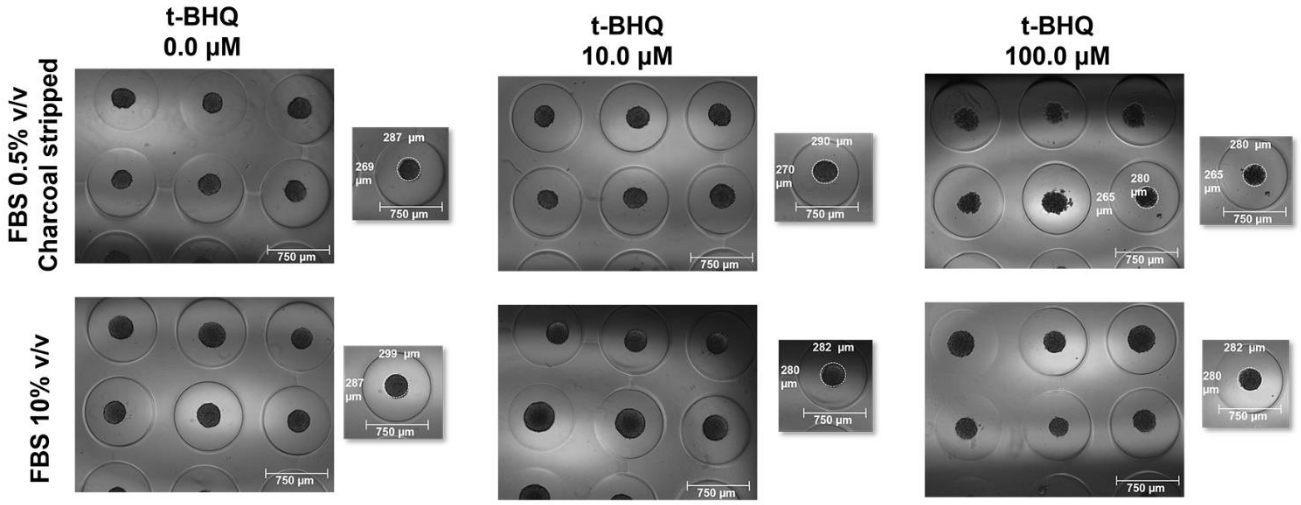

Figure S6: Brightfield images of 1 day-old HEK293T spheroids transfected with pGL4.37[luc2P/ARE/Hygro] and treated for 5 hrs with t-BHQ.

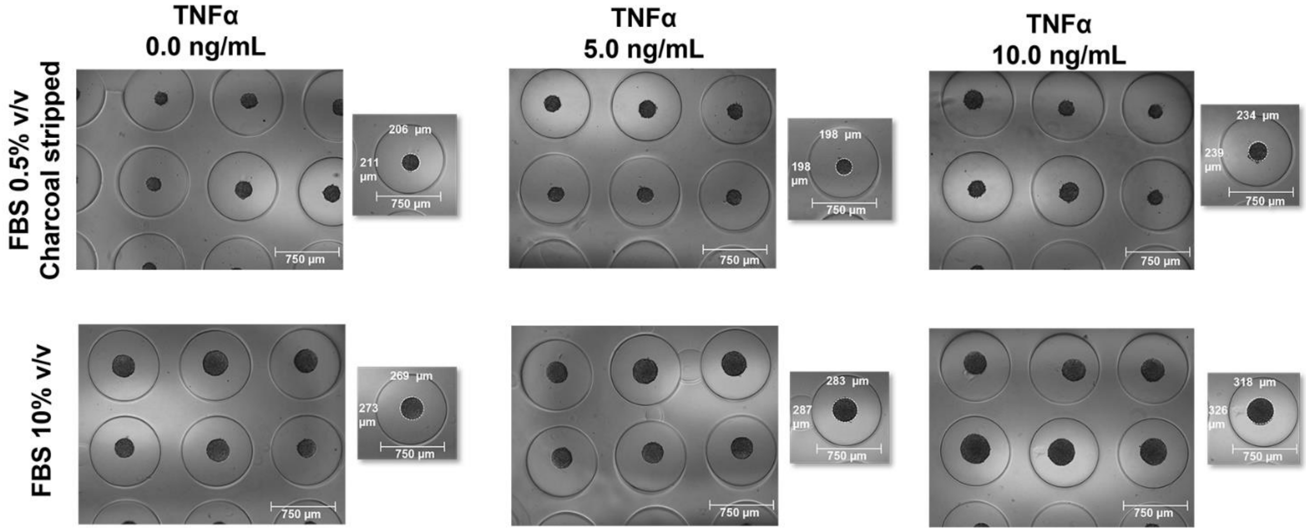

Figure S7: Brightfield images of 1 day-old HEK293T spheroids transfected with pGL4.32[luc2P/NF-κB-RE/Hygro] and treated for 5 hrs with TNFα.

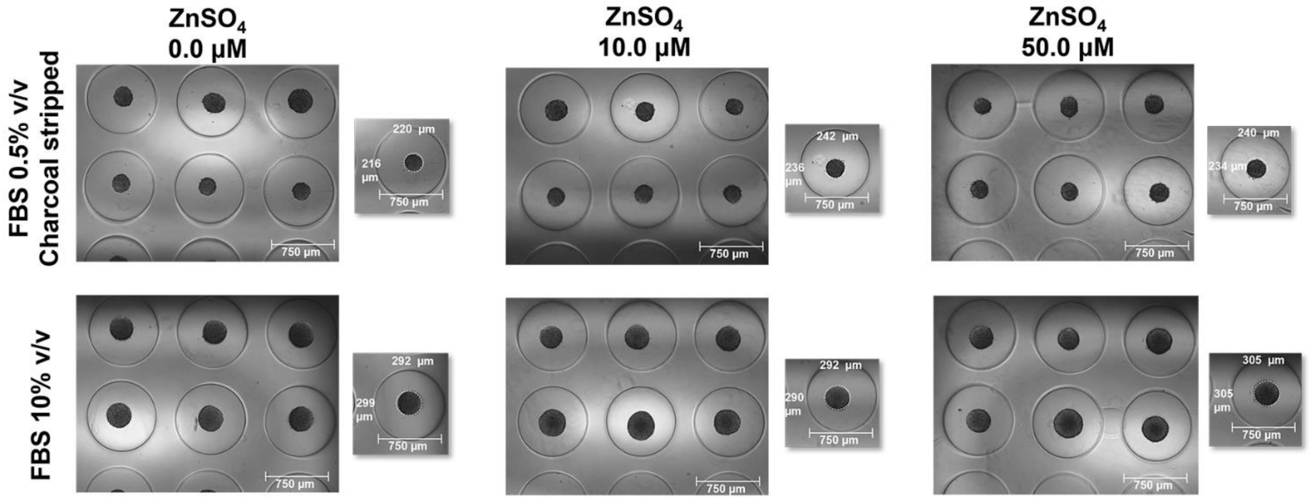

Figure S8: Brightfield images of 1 day-old HEK293T spheroids transfected with pGL4.40[luc2P/MRE/Hygro] and treated for 5 hrs with $ZnSO_4$.
